# A Vision-Based Procedure with Subpixel Resolution for Motion Estimation

**DOI:** 10.3390/s25103101

**Published:** 2025-05-14

**Authors:** Samira Azizi, Kaveh Karami, Stefano Mariani

**Affiliations:** 1 Department of Civil Engineering, University of Kurdistan, Sanandaj 6617715175, Iran; ka.karami@uok.ac.ir; 2 Department of Civil and Environmental Engineering, Politecnico di Milano, 20133 Milano, Italy; stefano.mariani@polimi.it

**Keywords:** vision-based identification, modal analysis, block matching, population-based optimization

## Abstract

Vision-based motion estimation for structural systems has attracted significant interest in recent years. As the design of robust algorithms to accurately estimate motion still represents a challenge, a multi-step framework is proposed to deal with both large and small motion amplitudes. The solution combines a stochastic search method for coarse-level measurements with a deterministic method for fine-level measurements. A population-based block matching approach, featuring adaptive search limit selection for robust estimation and a subsampled block strategy, is implemented to reduce the computational burden of integer pixel motion estimation. A Reduced-Error Gradient-based method is next adopted to achieve subpixel resolution accuracy. This hybrid Smart Block Matching with Reduced-Error Gradient (SBM-REG) approach therefore provides a powerful solution for motion estimation. By employing Complexity Pursuit, a blind source separation method for output-only modal analysis, structural mode shapes and vibration frequencies are finally extracted from video data. The method’s efficiency and accuracy are assessed here against synthetic shifted patterns, a cantilever beam, and six-story laboratory tests.

## 1. Introduction

Civil structures and infrastructures are continuously exposed to different hazards. Structural Health Monitoring (SHM) has therefore emerged as a necessary tool to predict possible structural failures and help prevent them [1]. As the local dynamic behavior of structures is sensitive to variations in their health, any deviation away from the normal or healthy condition may be exploited by the monitoring procedure. This is customarily performed by way of experimental modal analysis or operational modal analysis, which require the identification of relevant natural frequencies, mode shapes, and damping ratios of the monitored structure. Methods based on operational modal analysis process measurements collected through a network of sensors [2], which has to be deployed as widely as possible over the structure [3]. Even in the case of pavement monitoring, wireless sensor networks have been exploited to sense vibrations and estimate displacements/deformations induced by vehicles; see [4]. However, large-scale deployments are still a challenge, due to practical and cost-related constraints that can represent a barrier to the spread of related SHM strategies. Noncontact sensors, such as radar interferometers and laser vibrometers [5], are instead characterized by easier installation. On the other hand, they need a restricted measurement distance that prevents their use in case of large-scale structures [6].

In recent years, vision-based measurements have emerged as an effective method for full-field identification [7,8], damage detection [9,10], model updating [11], and motion estimation [12,13,14]. This novel method takes advantage of image data to obtain valuable insights into the structural behavior. By providing high-resolution response measurements, it also avoids any effect on the structural dynamics, due to, e.g., the weight of traditional sensing systems to be mounted on the structure, and also reduces the expenses related to the sensor network.

Block matching (BM) is one of the most effective motion estimation methods in digital video processing. With this method, images of a region of the structure under investigation (termed the block) are compared to a reference one. The structural motion is then obtained by minimizing the difference or by maximizing the cross-correlation between the current and the reference frames, within a predefined search area. The way this search for the best match is carried out represents a critical step of BM. A full search (FS) of all of the possible locations within the search area is highly effective but also highly expensive, featuring a computational complexity of $O(p^{2})$ , where p is the size of the search window. For real-time applications, to reduce the mentioned computational cost, algorithms like three-step search [15], diamond search [16], adaptive road pattern search [17], and fast full search [18] have been proposed. They all start with an initial evaluation at a few search points, to next refine the results based on the least block distortion. These methods often rely on fixed patterns and predefined limits of search, which can lead to errors in real-world cases where distortion varies unpredictably.

BM can be seen as an optimization problem, to be solved by approaches like differential evolution [19], genetic algorithms [20], or a population-based method [21], like Particle Swarm Optimization (PSO) [22], that improve the search efficiency and accuracy. Specifically, PSO has been shown to be very effective in solving the issue of distortion, linked to local minima of the objective function to be minimized. Thanks to the improvements provided by these algorithms, BM has been used also for micro/nano systems, aiming at displacement measurement for nano-positioning stages [23,24]. Its high precision in tracking motions has made it the preferred choice within vision-based measurement methods, also offering flexibility in handling complex motions of the structures [25,26]. It is now widely adopted in video coding for motion estimation and compensation [27,28], and to analyze temporal redundancy between frames, enabling video compression in standards such as H.264 and HEVC [29]. BM is also used in image registration to align images from different sources, particularly in fields like medical imaging [30] and remote sensing [31,32].

In relation to structural engineering, in [33], the displacements of a bridge were obtained by placing two LEDs on it, used as targets in the matching process. In [34], displacements were obtained by way of BM by maximizing the correlation when the target is artificial or part of the structure. Other methods were also proposed to extract features from the images that are invariant to brightness intensity and rotation [35,36,37], and finally lead to displacement time histories related to different collected frames. These methods are considered feature-based, and provide sparse measurements. In this direction, distinct regions of a six-story laboratory structure were studied in [38] and features were extracted [39]; subsequently, the Kanade–Lucas tracking algorithm [40] was exploited to obtain the displacements.

Other BM-based methodologies to estimate motions are digital image correlation [41,42,43], up-sampled cross-correlation, orientation code matching [44], and edge-enhanced matching [45]. BM techniques typically determine displacements at an integer pixel resolution, as the pixel represents the smallest unit in an image. Displacements at a subpixel resolution can be obtained by ways of curve fitting [46], Newton Raphson with an interpolation estimation [47,48], and gradient-based optical flow (GOF) [24,25,26,27,28]. The original optical flow technique was based on variational approaches, to obtain motion by solving equations within which assumptions regarding constant brightness patterns [24] or local phase [29,30] were made. In [49], it was demonstrated that the local phase of an image, obtained through quadratic filters [50], represents the motion more robustly than the intensity. By way of this, in [51], mode shapes were obtained from videos of vibrating structures, and they were subsequently employed to identify [52] and locate damage [53]. Motion estimation in a kind of hierarchical or multi-resolution framework, consisting of an integer pixel stage later refined at the subpixel scale, was originally introduced in [46] and expanded in [47], using a modified Taylor approximation. In [54], this two-stage strategy for targetless structures was studied by combining simple BM and phase-based optical flow in the horizontal direction. In [55], subspace identification was applied in different scenarios for healthy and simulated damage structures, with targets on the structural surface. As phase-based optical flow requires velocity-tuned filters proportional to the motion and a texture of the structural component to provide a one-dimensional motion estimation, an additional processing stage looks necessary to extract motion.

The aim of this work is instead to design an enhanced two-dimensional motion estimation framework for structural analysis, regardless of the vibration amplitude. The proposed solution exploits only the intensity for the motion estimation, within a multi-stage frame that integrates PSO-based BM at the pixel level and GOF at the subpixel one. The algorithm, overall, consists of four main steps in a coarse-to-fine framework: (1) Pre-evaluation of zero-motion pixels, to identify static blocks characterized by subpixel motion only and locally avoid the coarse-level estimation with PSO. (2) For the remaining locations, use of an efficient PSO-based BM and two adaptive strategies related to search limits, in place of the customary constant search limits, and pixel-subsampling blocks, in place of the fixed block size. (3) Use of GOF to achieve a fine-level motion estimation starting from that obtained at the previous stage. (4) Reduction of the estimation errors related to GOF and caused by noise in the case of small motions, through an error cancelation step. The proposed framework allows for attacking problems characterized by displacements either featuring a large or small amplitude, and provides robust solutions with lower computational costs than state-of-the-art alternate solutions. In summary, the proposed solution features the following novelties: (i) A hybrid automated coarse-to-fine algorithm adopted for two-dimensional structural motion estimation, without the need for specific targets to be placed on the structural surface. This framework combines the strengths of BM and gradient-based methods, further introducing enhancements to reduce errors and improve the robustness and efficiency of the estimation procedure; (ii) a blind modal analysis of the estimated motion using Complexity Pursuit (CP), for an in-depth assessment of the displacement accuracy aimed at modal identification.

The performance of the offered procedure is claimed to be independent of the motion magnitude, as is going to be validated on video data related to a synthetic shifted pattern, a lab-scale beam, and a six-story structure.

## 2. Smart BM and Reduced-Error Gradient Method

**Figure 1:**
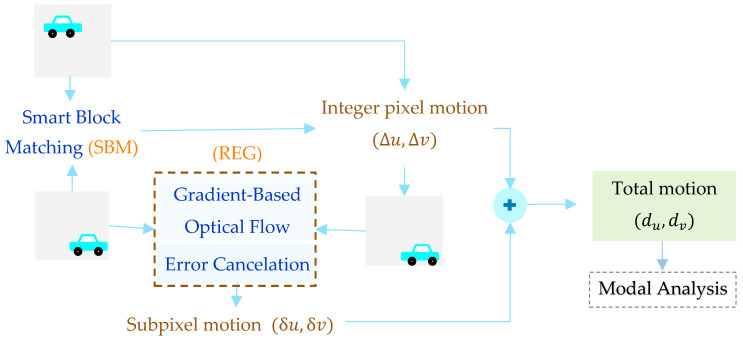
Workflow of the proposed method.

The algorithm for motion estimation is illustrated in the following. The workflow of the proposed Smart BM with Reduced-Error Gradient (SBM-REG) motion estimation is shown in Figure 1. To this aim, the two-dimensional structural motion $\left(d_{u},d_{v}\right)$ is partitioned into the integer pixel part ( ∆u,∆v) and the subpixel part (δu,δv) .

When the structural response is characterized by large motions, adaptive search limits appear necessary for a reliable estimation of the motion itself. First, within the BM strategy, the integer pixel motion is obtained using a stochastic search method, namely, PSO, for an efficient estimation without a pixel-by-pixel full search. An adaptive selection of the search area and a subsampling strategy for blocks of pixels, which is referred to as a Smart BM solution, lead to an accurate and robust motion estimation with low computational costs. Once the current frame is shifted by the estimated integer pixel motion (∆u,∆v) , just the subpixel motion (δu,δv) still has to be assessed. The latter is estimated by way of a GOF method, which is effective for guaranteeing accuracy in cases of small displacements; see [56]. Due to the said accuracy of the GOF method, the procedure does not require any interpolation between the intensity of the pixels. Although GOF is characterized by computational efficiency and simplicity, inaccuracies in the gradient computation can lead to biases in the estimation [40]. If accurate subpixel motion tracking is relevant, account must be taken of the fact that the bias is linearly proportional to the motion, so that the error increases with the magnitude of the actual shift [56]. By leveraging this relationship, an error cancelation step has been implemented to refine the GOF estimate, within an REG procedure. By next applying a blind source separation methodology on the extracted motion, modal parameters like modal shapes and frequencies can be estimated. This framework effectively balances the accuracy and computational efficiency, proving to be suitable for problems characterized by either large or small structural motions.

The remainder of this section is structured as follows: Section 2.1 provides details of the PSO-based BM method and of the updating strategy, while Section 2.2 introduces the GOF method along with the error cancelation step.

### 2.1. Enhanced Population-Based BM for Integer Pixel Motion Estimation

#### 2.1.1. Block Matching

**Figure 2:**
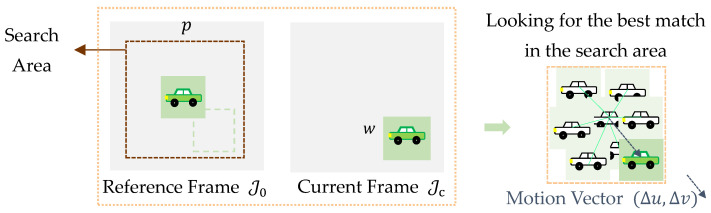
Sketch of the BM-based process.

BM is used to quantify the motion between a reference frame $J_{0}$ and the current frame $J_{c}$ of a video, under the assumption that the intensity of each pixel does not change significantly during motion. A schematic diagram of the process of BM measurement is shown in Figure 2. In it, the video is subdivided into frames and each frame is divided on its own into blocks, with the goal of tracking how each block moves from one frame to the next. A search area consisting of p×p pixels around the corresponding block of w×w pixels in the reference frame is selected to find the region that most closely resembles the initial block in the current frame. The similarity between the two blocks is determined through a criterion based on cross-correlation CC , according to

(1) $$CC\left(\Delta u,\Delta v\right)=\frac{\sum_{m=1}^{w}\sum_{n=1}^{w}\left[I_{0}\left(x_{m},y_{n}\right)-\bar{I_{0}}\right]\left[I_{c}\left({\overset{´}{x}}_{m},{\overset{´}{y}}_{n}\right)-\bar{I_{c}}\right]}{\sqrt{\sum_{m=1}^{w}\sum_{n=1}^{w}{\left[I_{c}\left({\overset{´}{x}}_{m},{\overset{´}{y}}_{n}\right)-\bar{I_{c}}\right]}^{2}}\sqrt{\sum_{m=1}^{w}\sum_{n=1}^{w}{\left[I_{0}\left(x_{m},y_{n}\right)-\bar{I_{0}}\right]}^{2}}}$$

where

(2) $${\overset{´}{x}}_{m}=x_{m}+du\;,\;\;\;{\overset{´}{y}}_{n}=y_{n}+dv$$

and $\left(x_{m},y_{n}\right)$ are the coordinates in a local block system, where the origin $\left(0,0\right)$ is the top-left corner; $I_{0}\left(x_{m},y_{n}\right)$ and $I_{c}\left({\overset{´}{x}}_{m},{\overset{´}{y}}_{n}\right)$ represent the gray (intensity) levels of the blocks in the reference and current frames, respectively; $\bar{I_{0}}$ and $\bar{I_{c}}$ are the mean values of the intensity of the block pixels, again in the reference and current frames. The displacement $\left(\Delta u,\Delta v\right)$ that maximizes CC represents the estimated motion of the block at the coarse scale. This process is repeated for all of the blocks, allowing motion estimation for the entire structure.

In traditional BM-based procedures, the search area and the block size are predetermined, and the best match is obtained by searching for all the possible locations within the search area, thereby resulting in being computationally intensive. Additionally, the fixed search limits become unreliable in the case of motions larger than the predefined limits, which therefore become case-dependent features. In SHM, cases are characterized by limited insight into the vibration amplitude at different locations, and a flexible framework for motion estimation appears to be essential. To tackle these challenge, in this work, an advanced motion estimation procedure is proposed. Starting from a pre-evaluation of the similarity at block location CC(0,0) to identify the zero-pixel motion based on a defined threshold th , static blocks and blocks featuring subpixel motion only are identified. For the remaining blocks, PSO is exploited to maximize CC given by Equation (1) together with two updating strategies to adjust the search area and perform sub-sampling of pixels within the blocks. The pre-evaluation step effectively prevents unnecessary analysis within the regions experiencing subpixel motion, while, in the case of larger motions, the framework dynamically expands the search space to ensure accuracy. Since CC ranges from 0 to 1 and images are typically affected by noise, the aforementioned threshold distinguishing the static blocks from the others is customarily set to 0.9 to point to blocks experiencing subpixel motion only. The chosen threshold represents a balance between computational efficiency and estimation reliability and does not critically affect the overall performance of the framework across different image noise levels or textures, as supported by our validation experiments.

#### 2.1.2. Particle Swarm Optimization

In the proposed solution, for each block, PSO departs from an initial guess of the solution to the maximization of CC , to iteratively refine it [57]. Regarding the notation, a particle is a candidate solution within a D -dimensional space based on the number of parameters to be optimized, while the swarm Z is formed by $n_{0}$ particles and provides the positions of all the particles in the said D -dimensional space. As the solution is sought in terms of the horizontal and vertical components of the motion $\left(\Delta u,\Delta v\right)$ , in the considered two-dimensional setting, D=2 one obtains the following:

(3) $$Z=\;\left\{{\left(z_{i1},z_{i2}\right)}_{i=1},{\left(z_{i1},z_{i2}\right)}_{i=2},\ldots ,{\left(z_{i1},z_{i2}\right)}_{i=n_{0}}\right\}$$

The initial locations $z_{i}(0)$ of the particles are randomly deployed with a uniform distribution within an interval upper and lower bounded by Ub and Lb , respectively. At the k -th iteration of the algorithm, k=1,…,Mi , to converge towards the optimal solution, the position of the i -th particle is updated according to

(4) $$z_{i}\left(k+1\right)=z_{i}\left(k\right)+v_{i}(k+1)$$

where $v_{i}$ is the velocity of the same particle, given by

(5) $$v_{i}\left(k+1\right)=\omega v_{i}\left(k\right)+c_{1}({pb}_{i}-z_{i}\left(k\right))r_{1}+c_{2}(gb-z_{i}\left(k\right))r_{2}$$

In Equation (5), ${pb}_{i}$ are the coordinates of the i -th particle related to the best solution obtained up to the k -th iteration; gb is the overall best solution obtained by the swarm up to the same k -th iteration; ω is the inertia weight; $c_{1}$ and $c_{2}$ are two acceleration constants, which take values in the range $\left[14\right]\;$ and control the motion of the particle within the current iteration, respectively, in the direction of its personal best ${pb}_{i}$ and of the global best gb ; $r_{1}$ and $r_{2}$ are two random variables, showing a uniform distribution in [0 1]. In this work, a linear decreasing function across the iterations has been adopted for ω , moving from ω=0.9 to ω=0.4 ; $c_{1}=2$ and $c_{2}=1$ have been selected instead [58]. The aforementioned best solutions provided by the particle and by the entire swarm are computed by way of the CC metric.

**Figure 3:**
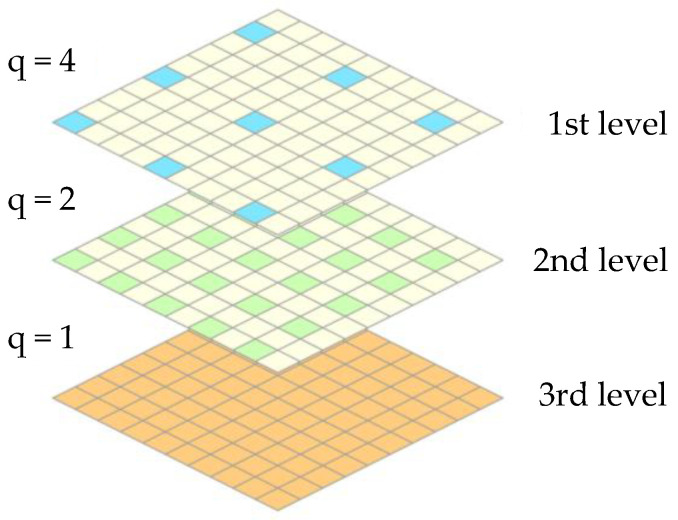
Sketch of three-level pixel subsampling of a block.

Thanks to pixel subsampling, the computational cost of the optimization process is reduced, as only some pixels in each block are used to compute the cross-correlation between the different frames. Moving from a block of size w , at the l -th algorithmic level of subsampling,  l= 1, 2,…,S , the selected pixels are spaced by ${q=2}^{S-l}$ additional pixels that are not allowed for in the cross-correlation computation; see Figure 3. With this subsampling strategy, the (subsampled) cross-correlation SCC is computed according to the following:

(6) $$SCC\left(∆u,∆v\right)=\;\frac{\sum_{b=0,m=1+bq}^{Ns}\sum_{e=0,n=1+eq}^{Ns}\left[I_{0}\left(x_{m}{,y}_{n}\right)-\bar{I_{0}}\right]\left[I_{c}\left({\overset{´}{x}}_{m},{\overset{´}{y}}_{n}\right)-\bar{I_{c}}\right]}{\sqrt{\sum_{b=0,m=1+bq}^{Ns}\sum_{e=0,n=1+eq}^{Ns}{\left[I_{c}\left({\overset{´}{x}}_{m},{\overset{´}{y}}_{n}\right)-\bar{I_{c}}\right]}^{2}}\sqrt{\sum_{b=0,m=1+bq}^{Ns}\sum_{e=0,n=1+eq}^{Ns}{\left[I_{0}\left(x_{m}{,y}_{n}\right)-\bar{I_{0}}\right]}^{2}}}$$

As *l* increases, more particles are accounted for in the evaluation of SCC , until the point that they are all considered at the S -th level. At each iteration, if SCC exceeds the given threshold th , the algorithm is stopped and the gb solution is considered as the final response to use as the initial guess for subpixel motion estimation. If the algorithm fails to find a solution attaining the threshold at the S -th level, the search boundaries are updated by increasing the size of the search space (by 20% in the current investigation) for the next generation G . In the results reported in this paper, S=3 and G=2 have been adopted.

**Figure 4:**
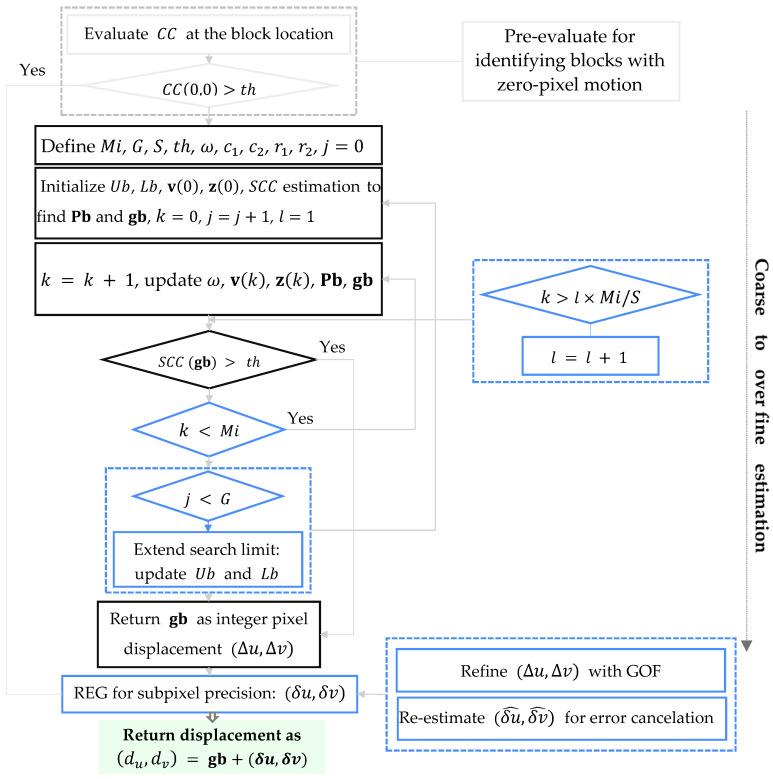
Flowchart of the SBM-REG method.

The flowchart of the entire SBM-REG procedure is shown in Figure 4.

### 2.2. Enhanced Gradient-Based Solution with Error Cancelation for Subpixel Motion Estimation

Once the coarse-level estimation (∆u,∆v) has been obtained, the block in the current frame is shifted accordingly toward the same block in the reference frame, and the estimation of subpixel motion (δu,δv) can be started on the fine scale. GOF [56] works by assuming a constant local intensity of a point in the blocks of the reference and current images, that is

(7) $$I_{0}\left(x+\delta u,y+\delta v\right)=I_{cr}\left(x,y\right)$$

Due to the subpixel displacement amplitude, $I_{0}$ is expanded in the Taylor series up to the first order in (δu,δv) , so that

(8) $$I_{0}\left(x+\delta u,y+\delta v\right)=I_{0}\left(x,y\right)+\delta u.\;{I_{0}}_{x}\left(x,y\right)+\delta v.\;{I_{0}}_{y}\left(x,y\right)$$

where ${I_{0}}_{x}$ and ${I_{0}}_{y}$ represent the spatial gradients of $I_{0}$ . Equation (8) can be solved to obtain the estimation $\left(\hat{\delta u},\hat{\delta v}\right)$ , using the least squares technique in the following form:

(9) $$\left[\begin{array}{c} \hat{\delta u}\\ \hat{\delta v} \end{array}\right]={\left[\begin{array}{cc} \sum_{x=1}^{w}\sum_{y=1}^{w}{\left({I_{0}}_{x}\right)}^{2} & \sum_{x=1}^{W}\sum_{y=1}^{W}\left({I_{0}}_{x}.{I_{0}}_{y}\right)\\ \sum_{x=1}^{w}\sum_{y=1}^{w}\left({I_{0}}_{x}.{I_{0}}_{y}\right) & \sum_{x=1}^{W}\sum_{y=1}^{W}{\left({I_{0}}_{y}\right)}^{2} \end{array}\right]}^{-1}\left[\begin{array}{c} \sum_{x=1}^{w}\sum_{y=1}^{w}({{I_{0}-I}_{cr})I_{0}}_{x}\\ \sum_{x=1}^{w}\sum_{y=1}^{w}{{{(I}_{0}-I}_{cr})I_{0}}_{y} \end{array}\right]$$

in which all of the terms are computed at coordinates (x,y) .

GOF is known to provide errors in the estimates, due to the gradient of image intensity, which increase with the magnitude of the motion or shift in the images [59]. Although the focus here is on subpixel motion estimation, an error cancelation procedure, termed REG, is adopted to obtain the fine-scale estimation. The solution $\left(\hat{\delta u},\hat{\delta v}\right)$ provided by Equation (9) is considered a linear transformation of the actual one $\left(\delta u,\delta v\right)$ , according to

(10) $$\left[\begin{array}{c} \hat{\delta u}\\ \hat{\delta v} \end{array}\right]=\left[\begin{array}{cc} \alpha_{1} & \alpha_{2}\\ \alpha_{3} & \alpha_{4} \end{array}\right]\left[\begin{array}{c} \delta u\\ \delta v \end{array}\right]$$

where the parameters $\alpha_{i}$ , i=1,2,3,4 , have to be set.

**Figure 5:**
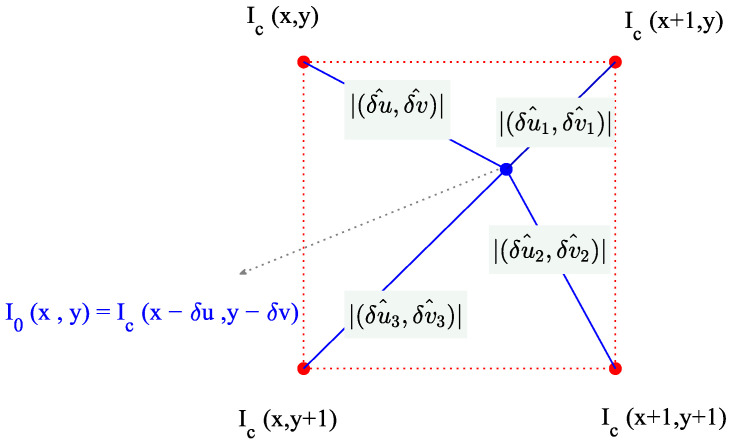
Additional subpixel shifts adopted for error cancelation purposes.

As the gradient estimation is obtained from the reference image, all of the possible shifts between different frames and the reference frame share the same parameters in Equation (10). By exploiting this assumption, the values of the parameters $\alpha_{i}$ can be estimated with additional synthetic shifts of the current frame; see Figure 5. Such additional ±1 -pixel shiftings have a direction opposite to that of the estimated values $\left(\hat{\delta u},\hat{\delta v}\right)$ . They allow for the estimation of the enhanced $\left(\delta u,\delta v\right)$ motion by shifting the image horizontally, vertically, and, finally, both horizontally and vertically, so that a slight redundancy in the equations is achieved in conjunction with Equation (10), to solve for $\alpha_{1},\alpha_{2},\alpha_{3},\alpha_{4}$ and $\left(\delta u,\delta v\right)$ .

By integrating REG into the proposed motion estimation procedure, in the Results Section, it is shown that the accuracy and reliability of the obtained measures are enhanced across the entire field, and the overall performance of the structural analysis is thereby improved.

## 3. Blind Modal Analysis with Complexity Pursuit

The video of the vibrating structure is assumed to be decomposed into T frames. As each frame is subdivided into N blocks, and the SBM-REG procedure is used to obtain the motion between the reference and the current frames for each block.

If the motions of all the blocks are collected in the matrix $D\in R^{N\times T}$ , an output-only modal analysis like blind source separation (BSS) [60,61] can be used to extract the relevant independent modal components. The structural motion D is accordingly expressed as a linear combination of the modal responses as

(11) $$D\left(X,t\right)=\phi \left(X\right)q\left(t\right)=\sum_{m=1}^{n}\varphi_{m}\left(X\right)q_{m}\left(t\right)$$

where t=1,2,..,T is a time-like variable ; X represents the block location, as defined by the coordinates of the center of the block; n is the number of excited modes; $\phi \in R^{N\times n}$ is the matrix collecting the vibration modes; $q\in R^{n\times T}$ are the modal responses. By using CP as a BSS technique, mode shapes and modal responses are obtained [62]; by next applying a Fast Fourier Transform (FFT) on the modal responses, the corresponding vibration frequencies are obtained.

CP leverages the temporal predictability of signals. It looks for a de-mixing (row) vector $w_{m}$ according to

(12) $$s_{m}\left(t\right)=w_{m}\;D(t)$$

and such that the recovered component $s_{m}$ possess a temporal structure simpler than the observed mixtures. The solution moves from the temporal predictability of a candidate signal $s_{m}\left(t\right)$ , measured through the following contrast function:

(13) $$F\left(s_{m}\right)=log\left(\frac{V(s_{m})}{U(s_{m})}\right)$$

where $V\left(s_{m}\right)$ captures the overall variability of $s_{m}$ while $U(s_{m})$ captures the local smoothness of $s_{m}$ , respectively defined as

(14) $$V\left(s_{m}\right)=\sum_{t=1}^{T}{\left(s_{m}\left(t\right)-{\bar{s}}_{m}\left(t\right)\right)}^{2}\;U\left(s_{m}\right)=\sum_{t=1}^{T}{\left(s_{m}\left(t\right)-{\hat{s}}_{m}\left(t\right)\right)}^{2}$$

and ${\bar{s}}_{m}$ and ${\hat{s}}_{m}$ are the moving averages:

(15) $${\bar{s}}_{m}\left(t\right)=\lambda_{L}\;{\bar{s}}_{m}\left(t-1\right)+\left(1-\lambda_{L}\right)\;s_{m}\left(t-1\right)\;{\hat{s}}_{m}\left(t\right)={\;\lambda }_{S}\;{\hat{s}}_{m}\left(t-1\right)+(1-\lambda_{S})s_{m}\left(t-1\right)$$

where $\lambda_{L}$ and $\lambda_{S}$ are parameters related to the long-term and short-term half-life.

By way of Equations (12), (14), and (15), Equation (13) can be written:

(16) $$F\left(w_{m},D\right)=log\left(\frac{V(w_{m},D)}{U(w_{m},D)}\right)=log\left(\frac{w_{m}\bar{R}{w_{m}}^{T}}{w_{m}\hat{R}{w_{m}}^{T}}\right)$$

where $\bar{R}\;$ and $\hat{R}$ are the covariance matrices related to the long-term and short-term variations of the displacement vector, respectively. In relation to the displacement matrix D(t) **,** CP allows one to solve the problem as an optimization one to determine the de-mixing vector $w_{m}$ by maximizing the temporal predictability contrast function F . For additional technical details, readers are referred to [62].

In what follows, the capability of the proposed method is assessed against synthetic patterns and laboratory test setups.

## 4. Experiments

### 4.1. Synthetic Shifted Patterns

**Figure 6:**
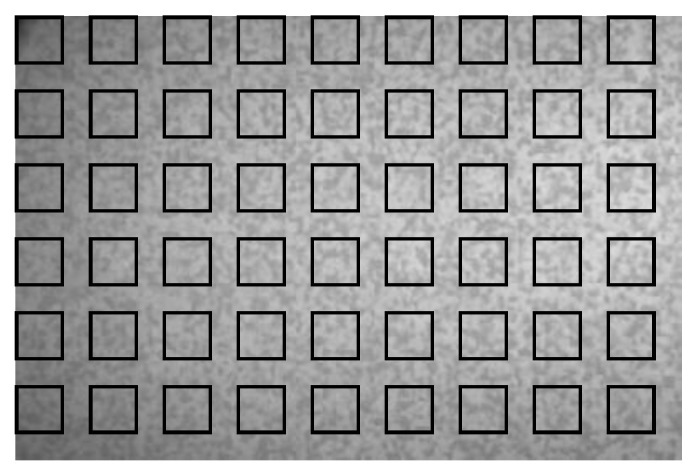
Synthetic shifted patterns: initial frame of sample 7 of the DIC challenge, with reported blocks for subpixel motion estimation.

Sample 7 of the DIC challenge proposed in [63] is considered as a first benchmark for the proposed solution. It consists of 12 speckle images featuring rigid-body subpixel translations ranging between 0 and 1 pixel, with a step of 0.1 pixel, in both the horizonal and vertical directions. The images have a size of 325×487 pixels, with contrast 100 and the noise variance 0.66; see also [64]. The first frame of this sample, featuring 54 blocks of 31×31 pixels, is shown in Figure 6.

**Figure 7:**
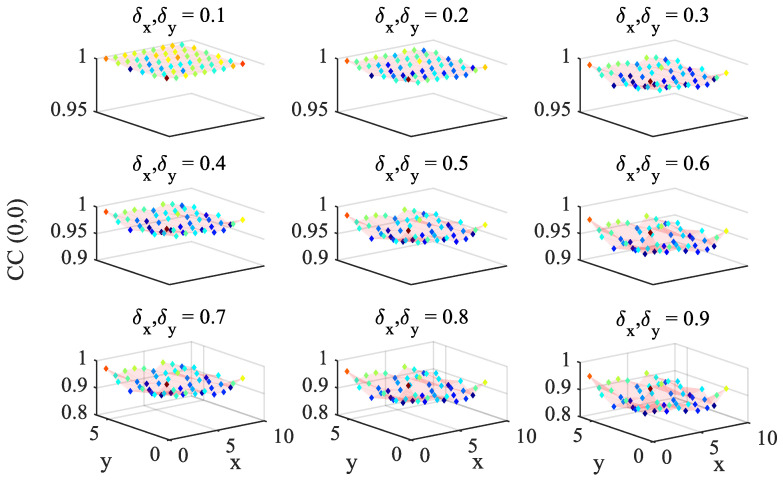
Synthetic shifted patterns: pre-evaluation of zero-motion cross-correlation CC at different block locations for different subpixel displacements.

Figure 7 collects results in terms of the cross-correlation CC , used to recognize the zero-motion at the block locations, for all of the frames. As expected, at lower subpixel displacements, the CC coefficient between the reference and the shifted images exhibits larger values. By setting a threshold to th=0.9 , six frames meet the condition for which the coarse estimation step is unnecessary and the REG-based solution can be directly adopted. Nevertheless, to enable a comparative analysis of GOF and REG methods, a threshold th=0.8 has instead been selected.

**Figure 8:**
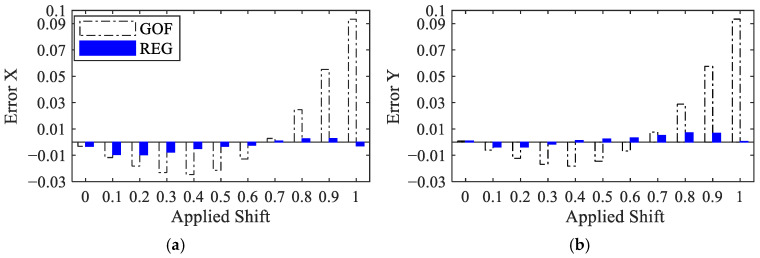
Synthetic shifted patterns: values of the estimation error provided by the GOF and REG methods, relevant to (**a**) horizontal and (**b**) vertical shifts (values in pixels).

To assess the accuracy of motion estimation, an error is computed as $(\frac{1}{a}\sum_{i=1}^{a}{∆}_{i})-{∆}_{applied}$ , where ${∆}_{applied}$ is the imposed subpixel displacement while ${∆}_{i}$ , i=1,…,a , are the estimated local values obtained with GOF at the different locations. This error measure is reported in Figure 8 for the horizontal and vertical components, and for all the steps of the applied shift. The GOF-based error value is shown to be negative for shifts in the range between 0 and 0.6 pixels, indicating that the method underestimates the imposed subpixel shift in both directions, with the error increasing for larger shifts. By applying the bias compensation procedure detailed in Section 2.2, the error in all the solutions is significantly reduced, even by a factor of ten for larger shifts.

It is notable that the estimation error is influenced by the spatial frequency of the frames, that is, by the rate of change of the intensity of pixels in the images. Additionally, different shifts in the two in-plane directions can affect the results and the relevant accuracy. Nevertheless, the objective of this study is only the assessment of the performance of the REG method in compensating the errors of the gradient method caused by noise (random bias) and by the gradient interpolator (systematic bias).

**Figure 9:**
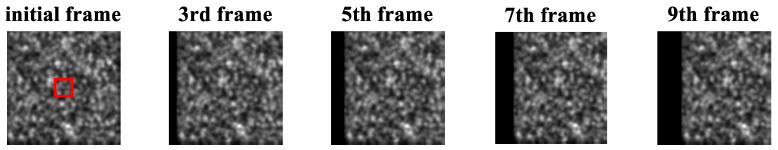
Synthetic shifted patterns: initial speckle pattern, and shifted frames with horizontal displacements for the SBM-REG performance evaluation.

**Figure 10:**
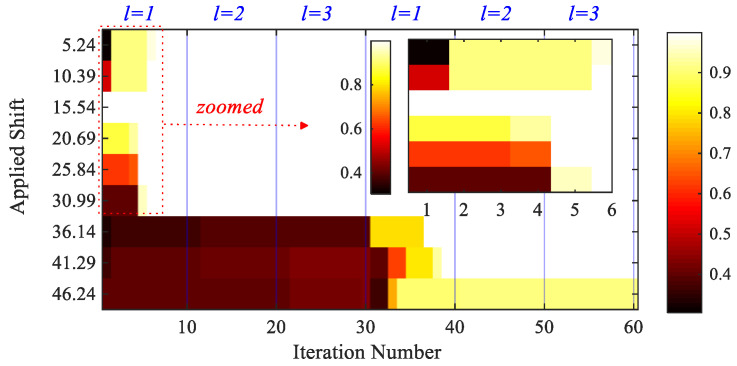
Synthetic shifted patterns: convergence of SCC scores across iterations, for different applied shifts.

Next, to assess the performance of the entire procedure, an 8-bit speckle pattern with a size of 200×200 pixels has been created as an initial frame [65], as shown in Figure 9. The subsequent frames showing horizontal motions have been obtained by applying displacements ranging from 5 to 50 pixels. A block of 31×31 pixels in the center of the frame is used to estimate the motion, with Mi= 21, 3 particles; th=0.92 ; initial search limits set to 30 pixels. Figure 10 shows how the SCC score changes with the iterations: for applied shifts smaller than 30 pixels, the estimation is completed in approximately six iterations at the first algorithmic level; for larger motions, a second generation is instead necessary to extend the search limits, and accurately estimate the motion.

Table 1 shows the estimated values of motion at the coarse, fine, and over-fine stages, along with the corresponding error index (EI) computed as the absolute difference between real and estimated values, divided by the real value. The SBM (coarse) estimation provides the integer part of the applied shift. As the estimations display, in all the cases with a one-pixel error, the selected threshold stops the algorithm properly. In fact, the same estimations are refined in the subsequent stages to attain a maximum error index of around 0.8%.

Even if not reported in detail for all the solutions, the ratio between the computation points of the FS and SBM-REG procedures shown at the bottom of Table 1 also highlights the computational efficiency of the offered method.

### 4.2. Cantilever Beam

**Figure 11:**
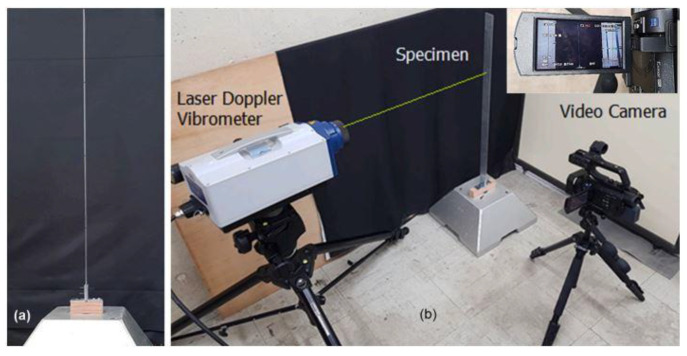
Cantilever beam: (**a**) initial configuration; (**b**) experimental setup featuring a video camera and a laser Doppler vibrometer (adapted from [66]).

The video of a vibrating beam (see [66]) is now considered. This video was captured by a camera with a frame rate of 30 fps, and the beam was initially deflected to induce free vibrations. Figure 11 shows the beam and the experimental setup aiming to simultaneously handle visual (camera-based) and laser measurements. The accessible video was reconstructed to display the beam vibrations related to its first mode, using phase-based motion magnification within the frequency range of 1–3 Hz.

**Figure 12:**
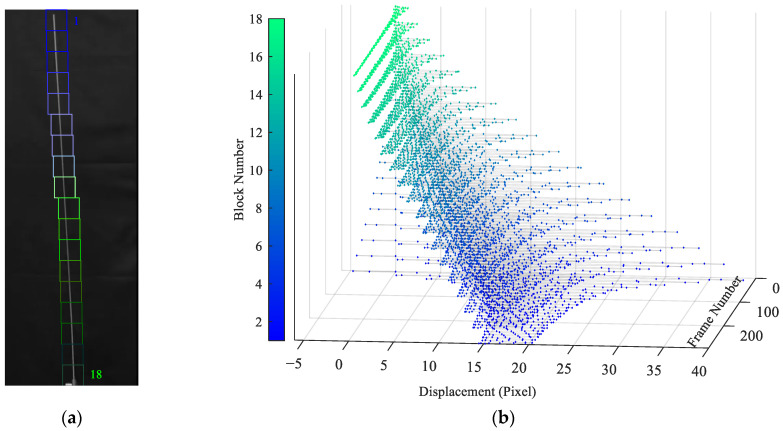
Cantilever beam: (**a**) reference frame captured by the video camera, and identified blocks along the longitudinal axis of the beam; (**b**) extracted motion of the 18 blocks in the horizontal direction.

The proposed algorithm has been adopted to extract the beam motion from the video, along the entire beam length. Figure 12a shows the reference frame of the video, whose size is 50×160 pixels. In the frame, 18 blocks of size 41×41 pixels are identified to extract the displacements. This procedure is at variance with the solution discussed in [66], characterized by 9 picks, as a denser solution is sought here. Since only the horizontal motion has to be extracted, the number of particles is set to 4, with Mi=42 and th=0.9 . The initial search limit is set to 40 pixels. Figure 12b displays the extracted displacements for all the blocks and the 293 considered frames.

**Figure 13:**
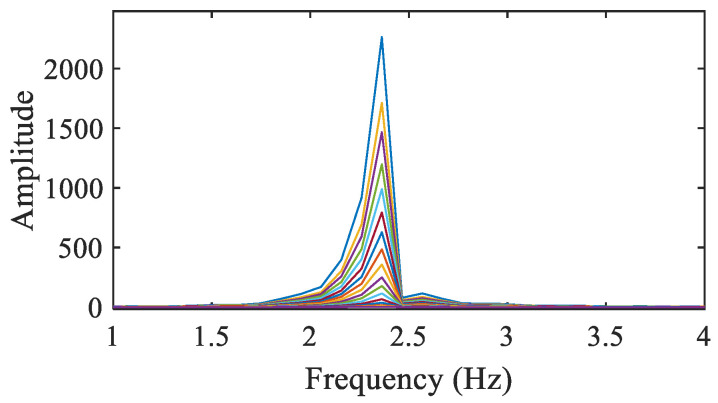
Cantilever beam: FFT of the extracted displacements at all block locations shown in Figure 12a. Different colors correspond to the block numbers 1 to 18 in Figure 12a.

By performing an FFT of the extracted displacements, see Figure 13, the excited vibration frequency of the beam is identified. The obtained frequency amounts to 2.363 Hz, which is in good agreement with the value of 2.43 Hz provided in [44]; the discrepancy between the two values is supposed to be related to the different framerates adopted in the two analyses.

**Figure 14:**
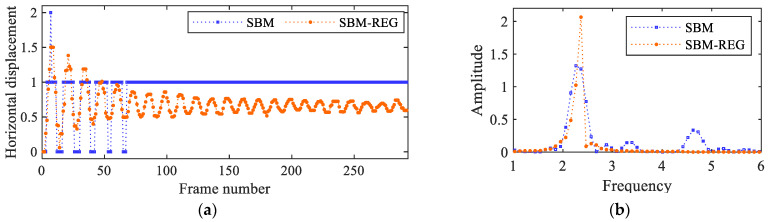
Cantilever beam: (**a**) extracted horizontal displacement time histories at the bottom of the beam, either allowing for subpixel precision or not; (**b**) corresponding FFTs of the signals.

The amplitude of the vibrations at the top of the beam is estimated to be about 40 pixels, while at the bottom of the beam, it is approximately 1 pixel. The SBM-REG procedure can therefore extract motions at different locations and with a noteworthy varying amplitude. To obtain insights into the benefit of the proposed multi-level procedure, Figure 14a shows a comparison between the extracted displacement time histories of the block at the bottom of the beam: here, integer pixel only and subpixel precisions are, respectively, associated with the SBM and SBM-REG solutions. The relevant FFTs are reported in Figure 14b: it can be seen that the integer pixel solution does not display a frequency peak as clear as the subpixel one, which leads to an estimated value of 2.363 Hz exactly as attained at the tip of the beam, where the magnitude of vibrations is at its maximum.

**Figure 15:**
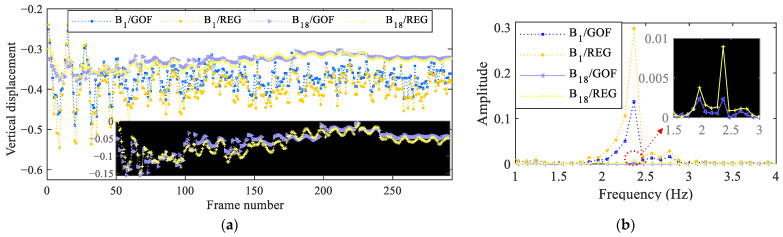
Cantilever beam: (**a**) extracted vertical displacement time histories at the bottom of the beam, either exploiting GOF or REG; (**b**) corresponding FFTs of the signals.

The discussion so far has been focused on the estimation of the horizontal displacements but, as the beam looks slightly deflected in its reference state, the vertical motion with subpixel precision can be studied as well. Figure 15a shows the estimated vertical motion obtained by allowing for zero-pixel motion through either GOF or REG, at the top and bottom of the beam. As seen in Figure 15a, the vertical motion estimated by REG at the top of the beam (B_1_/REG) is about 0.6 pixels, while that provided by GOF (B_1_/GOF) is about 0.4 pixels. The FFTs of the two signals, as shown in Figure 15b, also agree even if the spectrum provided by REG displays a clearer peak. The estimated motion at the bottom of the beam has an obvious smaller amplitude, such that a peak of the FFT given by GOF (B_18_/GOF) is not clear; the other way around, the estimation by REG (B_18_/REG) again shows a clear frequency peak at the correct value of vibrations.

To further assess the capability of the proposed SBM-REG method in tracking the two-dimensional motion of structures, the same cantilever beam has been considered and the handled video recording has been fictitiously rotated by $\theta =20^{0}$ . The results can therefore be compared with the former ones in terms of the amplitude of oscillations. In this analysis, parameters have been set as follows: 8 particles, Mi=45 , th=0.9 . The initial particle locations have been uniformly distributed around the relevant blocks, with a 30-pixel search limit.

**Figure 16:**
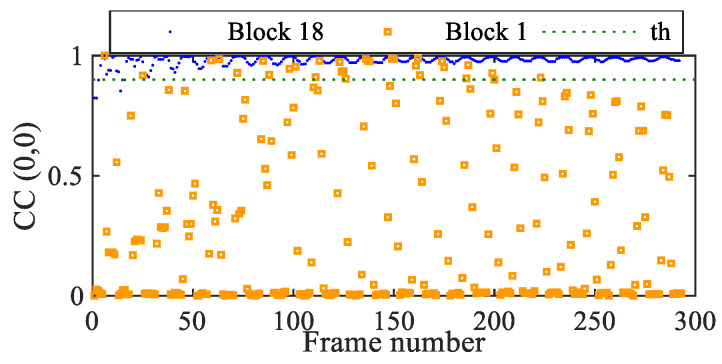
Cantilever beam: pre-evaluation of the cross-correlation at the top and bottom of the beam, for motion estimation with the rotated video.

Figure 16 depicts the outcome of the pre-evaluation step, at the top and bottom of the beam. It can be seen that, in almost all of the frames, the bottom block displays a value of CC larger than the threshold due to its subpixel motion. This means that extra processing linked to integer motion estimation with SBM is not necessary, and the subpixel part of the algorithm suffices to provide the entire solution. By way of this pre-evaluation step, the number of computational points is reduced by 99% for block 18 and by about 5% for block 1.

**Figure 17:**
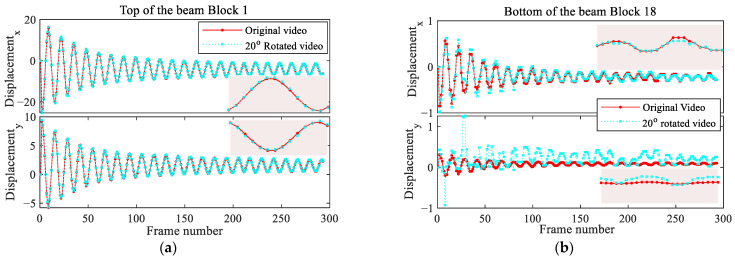
Cantilever beam: estimated motion at the (**a**) top and (**b**) bottom of the rotated beam.

The estimated horizontal and vertical components of the motion at the top and bottom of the rotated beam are compared with the projected original beam motion in Figure 17. While, at the top, the two solutions are in good agreement, at the bottom, the vertical components do not agree well. The reason for this discrepancy might be attributed to the applied rotation, which leads to a phase shift and therefore affects the results. The relevant FFTs, not reported here for brevity, provide, in all the cases, results in agreement with the former ones, with an estimated frequency of vibrations of 2.36 Hz at all the locations.

Finally, it is worth mentioning that the proposed solution only accounts for the intensity of pixels for BM, whereas existing subpixel estimation methods are phase-based and require the use of a directional filter for the extraction of the phase. Accordingly, the phase-based process leads to one-dimensional results while the proposed SBM-REG solution can provide estimations of two-dimensional motions.

### 4.3. Six-Story Structure

The six-story building model considered in [38] is now employed to study the effectiveness of blind modal analysis of multi degree-of-freedom structures; CP is adopted to process the extracted displacement field. If compared with the previous beam case, this structure is characterized by a wider frequency spectrum in its dynamics. A band-limited white noise excitation signal was adopted as input in the experiment to excite the different structural modes of vibration. The original video recording lasted 130 s and was taken by a camera recording at 30 fps, but that made available consists only of 50 s of video, comprising 1450 frames.

**Figure 18:**
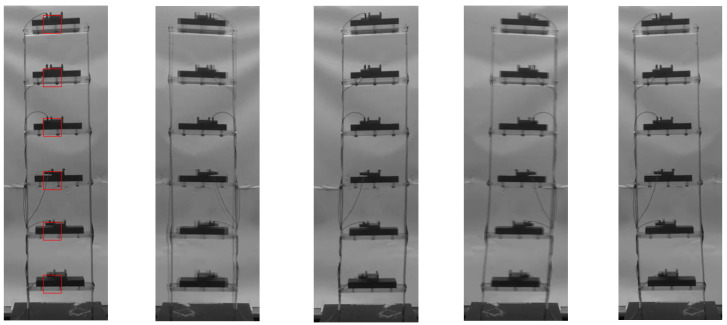
Six-story structure: five frames of the available video. The locations of the different blocks adopted in the analysis are shown in the first frame on the left.

Figure 18 shows five representative frames of this video, with the first frame on the left displaying the defined blocks, whose size is 41×41 pixels, defined to track the motion of each story. In terms of algorithmic parameters, the number of particles has been set to 6, with Mi=60 and the other PSO parameters set as before.

**Figure 19:**
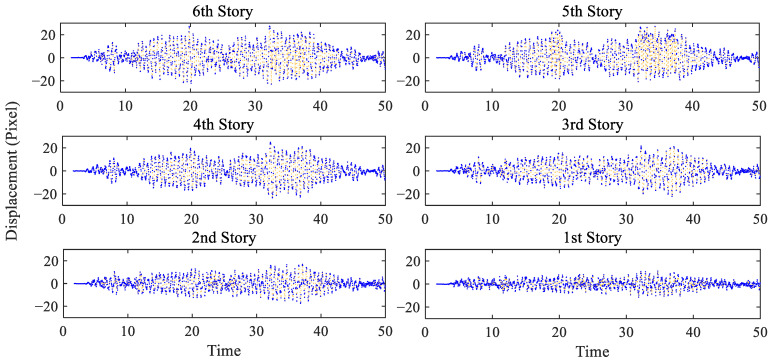
Six-story structure: story–displacement time histories extracted with the SBM-REG procedure.

**Figure 20:**
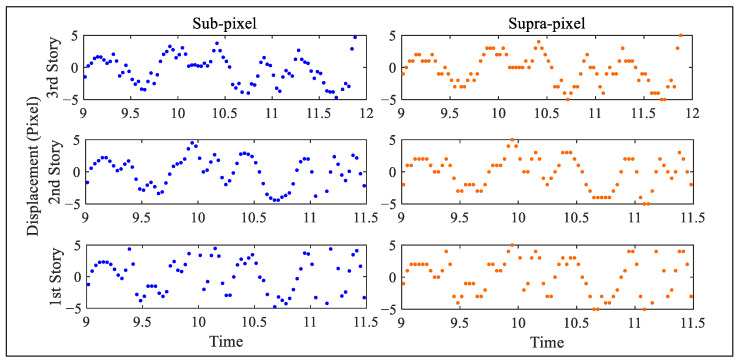
Six-story structure: comparison between the story–displacement time histories extracted with the (**left**) SBM-REG procedure, and the (**right**) SBM procedure.

Figure 19 reports the extracted displacements, measured again in pixels, for all of the stories. The maximum amplitude is clearly reported for the (top) 6th story, and amounts to 30 pixels, while the minimum one is related to the 1st story. The displacements obtained at the first level of the procedure have been obtained with a subset of 11×11 pixels, handled by PSO to converge within 14–17 iterations in all the solutions. To go more into the details of the efficacy of the proposed solution, the focus is next on the displacements of the bottom stories, which are characterized by smaller amplitudes and are therefore more in need of subpixel estimations. Figure 20 further illustrates the displacement time histories at the first, second, and third stories, by comparing the outcomes of the full SBM-REG procedure with subpixel resolution (left column), and those of the SBM procedure with integer pixel resolution only (right column). The qualitative improvement of the results is clearly visible, but it becomes even clearer by comparing the estimations of the vibration modes and frequencies.

**Figure 21:**
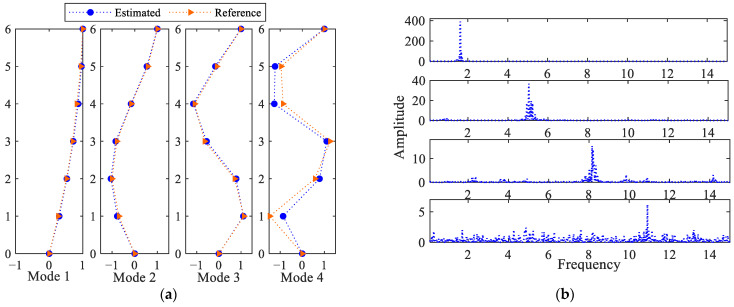
Six-story structure: (**a**) identified mode shapes, and (**b**) relevant FFTs.

Figure 21 collects the results in terms of the four fundamental modes identified by way of CP. The modes mentioned in [38] are also reported for a direct comparison with the current estimates. As shown in Figure 21a, the first three modes perfectly match the reference ones, while the fourth one shows a reasonably good agreement. The main reason for this difference could be related to the limited number of available frames, even if the solution is still considered accurate enough in overall terms. Figure 21b shows instead the FFTs of the extracted modal responses. The frequency peak values shown in the graphs are compared with the reference values in Table 2, to again show a close match between the values. 

**Figure 22:**
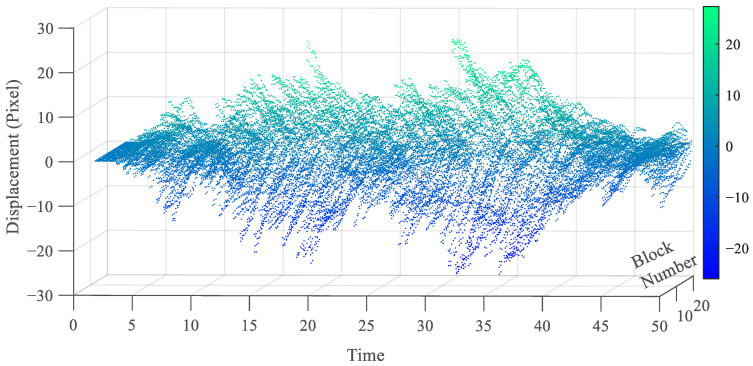
Six-story structure: full-field extracted motion of the 19 blocks in the horizontal direction.

**Figure 23:**
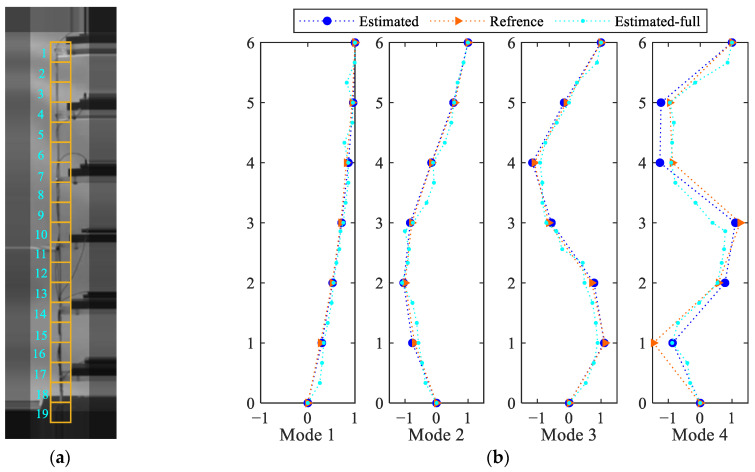
Six-story structure: (**a**) reference frame captured by the camera, and identified blocks along the left column of the structure; (**b**) identified mode shapes.

In general, the main goal of vision-based estimation techniques is to obtain full-field information on the vibrations of the structure under investigation. So far, the motion of the six stories has been investigated to compare the results with the data available in the literature. Next, the aim is moved to the estimation of the motion of a column of the building model in a full-field manner. This structural element is divided into 19 blocks, each with a size of 31×31  pixels. The estimated motions of all the blocks, as obtained with the proposed SBM-REG procedure, are shown in Figure 22. The coding for the blocks along the longitudinal axis of the structural element is shown in Figure 23a.

As the depicted results show a remarkable complexity and since modal analysis has to be performed for a number of measured locations that exceeds the number of excited modes, a Principal Component Analysis [51] is adopted first for order reduction purposes. Moving from the original motion matrix, which has size 19×1345 , by projecting the estimations onto the eigenvectors corresponding to eigenvalues that exceed 5% of the maximum one, and by finally exploiting CP, the full-field mode shapes can be estimated and are shown in Figure 23b. The results are compared in the charts with the previous estimations and the reference data, to stress that the extracted modes are in good agreement with those obtained with discrete measurements at story levels. The extracted frequencies, termed in Table 2 as ‘Estimated-Full’, match the reference frequency of the second mode of vibration well and show bounded differences for the other ones, though slightly worse than those obtained with blocks placed exactly at the different stories. Such a result points toward the issue of a proper design of the monitoring system, to assure that the deformation modes can be accurately recognized through the acquired measurements.

## 5. Conclusions

In this paper, a new video-based processing method for two-dimensional estimations of the motion of civil structures has been proposed. The main goal being system identification of modal parameters, the solution is foreseen to provide a means for future applications related to SHM. The offered Smart BM with Reduced-Error Gradient (SBM-REG) approach combines the strengths of BM and optical flow methods, to achieve accurate motion estimations independently of the vibration amplitude. By employing a coarse-to-fine-scale strategy, the framework integrates enhanced PSO-based BM for coarse-level motion estimation with gradient-based optical flow for fine-level estimations, in a kind a multi-resolution strategy.

Key enhancements to tackle the current challenges are as follows: the exploitation of a stochastic search method, to avoid becoming entrapped into local suboptimal solutions in terms of motion estimation; a strategy for the motion estimation characterized by adaptive search limits, to make the solution more robust; a pixel subsampling strategy with a relevant cost function, to reduce the overall computational burden; and an error cancelation strategy, to remove the systematic error of the gradient-based method. Based on the reported validation outcomes, the proposed SBM-REG framework has been shown to extract displacement time histories with subpixel resolution based on the image intensity only, thereby making it possible to avoid the use of targets or patterns on the monitored structure. The integration of the Smart BM and of the Reduced-Error Gradient-based method provides a capability to deliver accurate motion estimations also for cases characterized by large and small amplitudes of vibration in different regions of the same structure. Hence, the proposed framework lays a foundation for advanced motion estimations and vision-based structural dynamic analysis based on blind modal identification techniques.

It should be noted that the reliability of gradient-based estimation may decrease in low-texture regions or under motion blur, making it necessary to detect unreliable regions as part of a full-field analysis. This limitation will be addressed in future developments by integrating a detection strategy to identify and manage such regions.

Future works will also focus on deformation measurements, which require a six-dimensional optimization frame within PSO, and a Newton–Raphson technique for adaptive refinement. Furthermore, the linear models used in REG will be used to estimate the motion of neighboring blocks and by next leading to damage detection, though a comparison between the estimated motion related to a healthy solution and the actual one observed in the video data, moving towards a digital twin to be adopted in SHM strategies.

## Figures and Tables

**Table 1:** Synthetic shifted patterns: comparison of shift estimation accuracies at the different procedure stages.

Applied Shift	5.24	10.39	15.54	20.69	25.84	30.99	36.14	41.29	46.44
SBM (coarse) EI %	6 14.5	10 3.75	16 2.9	21 1.49	26 0.6	30 3.19	36 0.38	41 0.7	45 3.1
SBM-GOF (fine) EI %	5.1584 1.67	10.4525 0.6	15.4765 0.41	20.6465 0.21	25.8196 0.08	31.1679 0.57	36.1450 0.013	41.3357 0.1	46.9772 1.16
SBM-REG (over fine) EI %	5.2494 0.18	10.3964 0.006	15.5454 0.034	20.6853 0.022	25.8456 0.021	31 0.032	36.1450 0.013	41.3003 0.0249	46.8029 0.78
FSF/SBM-REG calculation points	5.83	13.35	85	22.02	27.03	25.86	-	-	-

**Table 2:** Six-story structure: identified modal frequencies, and comparison with the reference values reported in [38] (values in Hz).

	Mode 1	Mode 2	Mode 3	Mode 4
Reference	1.657	5.038	8.138	10.833
Estimated Estimated-Full	1.644 1.631	5.045 5.045	8.167 8.208	10.91 10.64

## Data Availability

The raw data supporting the conclusions of this article will be made available by the authors on request.
